# Ramoplanin at Bactericidal Concentrations Induces Bacterial Membrane Depolarization in Staphylococcus aureus

**DOI:** 10.1128/AAC.00061-14

**Published:** 2014-11

**Authors:** Mu Cheng, Johnny X. Huang, Soumya Ramu, Mark S. Butler, Matthew A. Cooper

**Affiliations:** Institute for Molecular Bioscience, The University of Queensland, Brisbane, Queensland, Australia

## Abstract

Ramoplanin is an actinomycetes-derived antibiotic with broad-spectrum activity against Gram-positive bacteria that has been evaluated in clinical trials for the treatment of gastrointestinal vancomycin-resistant enterococci (VRE) and Clostridium difficile infections. Recent studies have proposed that ramoplanin binds to bacterial membranes as a C_2_ symmetrical dimer that can sequester Lipid II, which causes inhibition of cell wall peptidoglycan biosynthesis and cell death. In this study, ramoplanin was shown to bind to anionic and zwitterionic membrane mimetics with a higher affinity for anionic membranes and to induce membrane depolarization of methicillin-susceptible Staphylococcus aureus (MSSA) ATCC 25923 at concentrations at or above the minimal bactericidal concentration (MBC). The ultrastructural effects of ramoplanin on S. aureus were also examined by transmission electron microscopy (TEM), and this showed dramatic changes to bacterial cell morphology. The correlation observed between membrane depolarization and bacterial cell viability suggests that this mechanism may contribute to the bactericidal activity of ramoplanin.

## INTRODUCTION

**FIG 1:**
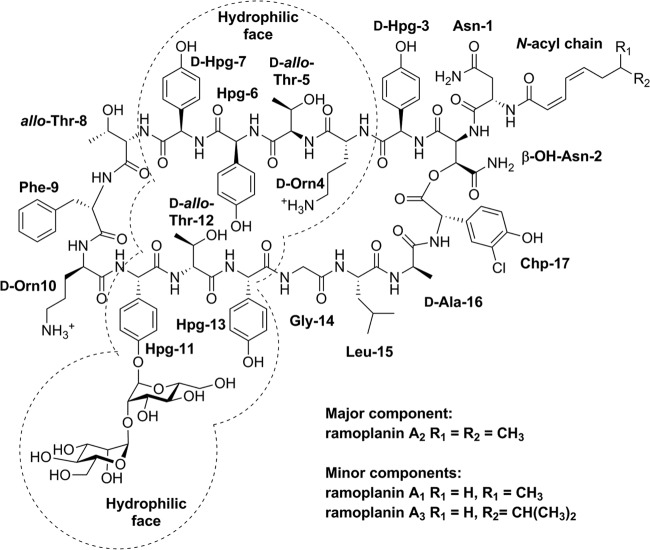
Structures of ramoplanin A_1_, A_2_, and A_3_, showing amino acid positional assignments and with the hydrophilic face (26) highlighted.

Ramoplanins A_1_, A_2_, and A_3_ (Fig. 1) are produced by Actinoplanes sp. ATCC 33076 and slightly differ in their lipid substituents (1, 2). These lipoglycodepsipeptide antibiotics disrupt cell wall biosynthesis (3–7) and possess potent activities against Gram-positive bacteria, including methicillin-resistant Staphylococcus aureus (MRSA), Staphylococcus epidermidis, streptococci, vancomycin-resistant enterococci (VRE), Bacillus spp., Listeria monocytogenes, and the anaerobe Clostridium difficile (8–11). Ramoplanin is bactericidal at concentrations close to its MIC (12), in contrast to vancomycin, which is bacteriostatic near its MIC. In 2006, Oscient Pharmaceuticals evaluated orally dosed ramoplanin, which is not systemically absorbed, in late-stage trials for the treatment of Clostridium difficile-associated diarrhea (CDAD) and VRE gastrointestinal colonization (13–16). Despite being administered intravenously to mice (17, 18), rats (17, 18), and rabbits (19) in *in vivo* models, parenteral administration of ramoplanin in humans is complicated due to hemolysis (13, 18, 20) and loss of activity due to hydrolysis of the depsipeptide ester (14). Nano Therapeutics, Inc., acquired the rights to develop ramoplanin in 2009 and recently announced that a phase IIb trial has been scheduled for September 2014 to investigate the use ramoplanin (coded NTI-851) as a targeted prophylaxis for recently treated patients with C. difficile infection (CDI) at high risk for infection relapse (21).

An early mode-of-action study proposed that ramoplanin inhibited the intracellular glycosyltransferase (MurG)-catalyzed conversion of Lipid I (undecaprenyl-pyrophospho-*N*-acetylmuramyl-pentapeptide) to Lipid II (undecaprenyl-pyrophospho-*N*-acetylmuramyl-*N*-acetylglucoseamine-pentapeptide) (3, 4), but later studies by Walker and coworkers showed that ramoplanin instead blocked the transglycosylation step of peptidoglycan biosynthesis by interfering with the transglycosylase-catalyzed extracellular polymerization of Lipid II (5–7). A good correlation between the ability of ramoplanin to inhibit transglycosylation *in vitro* (22) and its MICs against most Gram-positive strains supports this mechanism as its primary mode of action (14). Additional support for Lipid II as the primary target of ramoplanin is its peripheral location on cell membranes, while Lipid I is found exclusively on the cytoplasmic side of cell membranes (14). Also, ramoplanin is unlikely to readily penetrate the bacterial membrane due to its large size (molecular mass, 2,554 Da) and aqueous solubility (>100 mg/ml) (14, 23).

Ramoplanin contains an *N*-acyl chain linked to Asn-1 (Fig. 1) (10, 14) which may insert into the bacterial membrane phospholipid bilayers, as is the case for the lipoglycopeptide teicoplanin (24, 25). Recent studies have proposed that ramoplanin forms an intimate and highly amphipathic dimer in the membrane environment and binds to bacterial membranes via its hydrophobic interface (6, 26). In addition, the two positively charged ornithines (Orn) at positions 4 and 10 (27) may also interact with anionic phospholipids that predominate in Gram-positive bacteria (28, 29). Evidence for the membrane association of ramoplanin was published by McCafferty and coworkers, who reported that ramoplanin bound to phosphatidylethanolamine (PE) and phosphatidylglycerol (PG) phospholipid unilamellar vesicles in the absence of Lipid II, but no experimental details were provided (26).

In this study, a dose-dependent membrane association of ramoplanin was supported by results of surface plasmon resonance (SPR) studies that also showed a propensity to bind preferentially to anionic over zwitterionic membranes. Membrane effects of ramoplanin on methicillin-susceptible S. aureus (MSSA) were assessed by using a combination of a membrane depolarization assay and transmission electron microscopy (TEM) examination of bacterial ultrastructures. Ramoplanin was shown to dissipate the membrane potential at concentrations close to or above the minimal bactericidal concentration (MBC), which was consistent with its rapid bactericidal activity against the MSSA strain ATCC 25923.

## MATERIALS AND METHODS

### Antibiotics, bacterium, media, and phospholipids.

Ramoplanin containing >75% ramoplanin A_2_, vancomycin hydrochloride hydrate salt, teicoplanin containing >80% teicoplanin A_2_, and nisin from Lactococcus lactis were purchased from Sigma-Aldrich (Sydney, Australia). Citropin 1.1 (GLFDVIKKVASVIGGL-NH_2_) was purchased from Chiron Mimotopes (Melbourne, Australia). MSSA ATCC 25923 was purchased from the American Type Culture Collection (Manassas, VA). Mueller-Hinton broth (MHB; Bacto Laboratories Pty. Ltd.) was used to grow MSSA for determination of *in vitro* antibacterial activity in the presence and absence of 50% human serum (Life Technologies Australia, Melbourne, Australia) and time-kill assays. Luria-Bertani (LB) medium (Difco) and MHB adjusted with Ca^2+^ (25 mg/liter) and Mg^2+^ (12.5 mg/liter) (CA-MHB) were used for bacterial growth in the membrane depolarization assay and TEM, respectively. The phospholipids used for SPR analysis, 1-palmitoyl-2-oleoyl-*sn*-glycero-3-phosphoglycerol (POPG), 1-palmitoyl-2-oleoyl-*sn*-glycero-3-phosphocholine (POPC), and 1-palmitoyl-2-oleoyl-*sn*-glycero-3-phosphoethanolamine (POPE), were purchased from Auspep Pty. Ltd. (Melbourne, Australia).

### Antibacterial activity.

MICs against MSSA ATCC 25923 were measured by broth microdilution according to the Clinical and Laboratory Standards Institute (CLSI) M7-A7 methodology (30). Briefly, serial 2-fold dilutions of each antibiotic were added into Costar nontreated polystyrene 96-well plates, and each well was inoculated with 50 μl of MSSA in MHB to a final concentration of approximately 5 × 10^5^ CFU/ml. The MIC was the lowest antibiotic concentration that showed no visible growth after 24 h of incubation at 37°C. The dilution representing the MIC and two of the more and less concentrated dilutions were plated out onto Trypticase soy agar plates and enumerated to determine viable CFU/ml (31). The MBC was the lowest concentration of antibiotic yielding a 99.9% reduction in the initial colony count after 24 h of incubation.

### Time-kill studies.

Time-kill studies were carried out based on guideline M26-A of the CLSI (32), using Costar nontreated polystyrene 96-well plates. The kill kinetics of ramoplanin against MSSA ATCC 25923 were tested by incubating an initial inoculum of approximately 5 × 10^5^ CFU/ml with drug concentrations at the MIC, two dilutions above the MIC (2× and 4× MIC), and one dilution below the MIC (0.25× MIC) in MHB. Culture aliquots were mixed with the charcoal suspension (25 mg/ml) to minimize antibiotic carryover. Viable cell counts were determined after 0, 0.5, 1, 2, 4, 6, 8, and 24 h of incubation at 37°C by plating serially diluted samples onto Trypticase soy agar plates. Bactericidal activity was defined as a ≥3-log_10_ CFU/ml decrease, in comparison with the baseline, after 24 h of incubation (32). Vancomycin was used as a control, with drug concentrations at 2×, 1×, and 0.5× MIC. The time-kill assays were performed twice independently, with similar results.

### Liposome preparation.

Lipid stock solutions were prepared in chloroform and mixed at the described mass composition of POPC, POPC/POPG (8:2, wt/wt) and POPC/POPE (8:2, wt/wt) (33), and the chloroform was evaporated as previously described (34). The lipids were resuspended in 1 ml of filtered (0.22-μm pore size) running buffer (phosphate-buffered saline [PBS]; pH 7.3) and then sonicated for 25 min in 5-min intervals. Small unilamellar vesicles (SUVs) were prepared by extrusion through a polycarbonate filter with a 50-nm pore diameter (35).

### SPR.

SPR experiments were carried out with a Biacore T200 system (GE Health, Australia) using a Biacore vesicle capture (L1) sensor chip, which has lipophilic groups covalently attached to carboxymethylated dextran that facilitates direct lipid bilayer deposition (34, 36). All measurements were undertaken at a temperature of 25°C to maintain the lipid bilayer fluidity (34). SUVs at a total lipid concentration of 0.5 mg/ml were immediately passed across the chip surface for 60 min at a low flow rate of 2 μl/min, following three 30-s injections of 20 mM 3-cholamidopropyl-dimethylammonio-1-propane sulfonate (CHAPS) solution at a high flow rate of 30 μl/min to completely remove the captured vesicles from the sensor chip. To remove any multilamellar structures from the lipid surface and to stabilize the baseline, 10 mM NaOH was injected for 1 min at 50 μl/min. The coverage extent of the surface was later determined by a 5-min injection of 0.1 mg/ml bovine serum albumin (BSA) at a flow rate of 10 μl/min.

The antibiotics were serially diluted in running buffer and then injected sequentially from the lowest (0.01 μM) to the highest (10 μM) concentration at a flow rate of 20 μl/min for 180 s, followed by a dissociation of 300 s and a 1-min regeneration with 10 mM HCl at a flow rate of 10 μl/min. SPR experiments were performed in triplicate. The actual amount of antibiotic bound to each lipid bilayer was corrected by subtraction of the bulk refractive index difference (buffer control) and then normalized by dividing the antibiotic-bound resonance units (RU_Bound_) obtained in the SPR sensorgrams (see Fig. 3A, below) by the corresponding antibiotic molecular weight and the resonance units of the individual lipid vesicle captured on the chip surface (RU_Lipid_) (see Fig. S1 in the supplemental material) by using the following equation: normalized antibiotic bound = (10^3^ × RU_Bound_)/(molecular weight × RU_Lipid_). Normalization against molecular weight and RU_Lipid_ was necessary, as SPR units are mass dependent and RU_Lipid_ values vary according to lipid type (37).

### Membrane depolarization assay.

Antibiotic-induced bacterial cytoplasmic membrane depolarization was determined by using the fluorescent dye 3,3-dipropylthiacarbocyanine [DiSC_3_(5); Sigma-Aldrich, Australia] as previously described (38) with a high-throughput modification. Briefly, mid-logarithmic-phase MSSA ATCC 25923 cells were collected by centrifugation (5,000 × rpm, 10 min), washed once and diluted to approximately 5 × 10^7^ CFU/ml in buffer (5 mM HEPES, 5 mM glucose; pH 7.2). The cell suspension was incubated with 0.4 μM DiSC_3_(5) until dye uptake was maximal, as indicated by a stable reduction in fluorescence of assay medium. KCl at 100 mM was added to equilibrate the cytoplasmic and external K^+^ concentrations. A 90-μl aliquot of cell suspension was transferred into an Optiplate 96-well white microplate (PerkinElmer Corp., Australia), and 10 μl of antibiotic was added, to yield a series of solutions ranging from 0.1 to 30 μM. A blank with only cell suspension and dye was used for background subtraction. The fluorescence intensity was monitored in real time by using a BMG Labtech PolarStar Omega multimode reader fitted with 620-10 and 665-10 excitation and emission filters, respectively, at an excitation wavelength of 622 nm and an emission wavelength of 670 nm. The membrane depolarization assays were performed in triplicate, and the fluorescence leakage (F_L_) was defined by the following equation: F_L_ = (F_F_ − F_B_) − (F_I_ − F_B_). Here, F_F_ was the final fluorescence intensity in assay medium after 30 min of treatment with antibiotic, F_I_ was the initial fluorescence intensity of the cell suspension, and F_B_ was the fluorescence intensity of the blank. Citropin 1.1 induces complete and stable fluorescence leakage at 10 μM (∼0.5× MIC against MSSA ATCC 25923) (37) and was used to normalize the membrane depolarization of other antibiotics using the following formula: normalized membrane depolarization (as a percentage) = F_L(Antibiotic)_/F_L(Citropin 1.1 at 10 μM)_ × 100%.

### TEM.

Preparation and examination of ramoplanin- and vancomycin-treated MSSA ATCC 25923 cells by TEM were performed as described previously (39, 40). Exponential-phase bacteria in CA-MHB were exposed to ramoplanin at 1× MIC or vancomycin at 16× MIC for 3 h at 37°C. After centrifugation (8,000 × rpm, 3 min), the pellets were resuspended in 1 ml of 3% (vol/vol) glutaraldehyde in 0.1 M sodium cacodylate. Glutaraldehyde–fixed samples were washed twice with 0.1 M sodium cacodylate. To postfit cells, 1% (wt/vol) osmium tetraoxide was added. After the wash step, samples were stained with 2% (wt/vol) uranyl acetate in 50% ethanol. Samples were then dehydrated with graded ethanol solutions and infiltrated with Epon resin (ProSciTech, Townsville, Australia). All processes were performed on a Pelco 34700 Biowave microwave oven (Ted Pella Inc., Redding, CA). Ultrathin sections were cut at 60 to 70 nm by using a UC6 ultramicrotome (Leica). The sections were examined in a JEM 1011 TEM operated at 80 kV and photographed using a digital camera.

## RESULTS

### Antibacterial activity.

Ramoplanin displayed an MIC and an MBC against MSSA ATCC 25923 of 2 and 4 μg/ml, respectively, consistent with the published MIC and MBC ranges (41–44). The comparator antibiotics possessed the following MICs: vancomycin (1 μg/ml), teicoplanin (1 μg/ml), nisin (32 μg/ml), and citropin 1.1 (32 μg/ml). The MICs of ramoplanin and vancomycin in the presence of 50% human serum were 0.5 and 2 μg/ml, respectively (see Table S1 in the supplemental material).

### Ramoplanin is rapidly bactericidal at the MBC and above.

**FIG 2:**
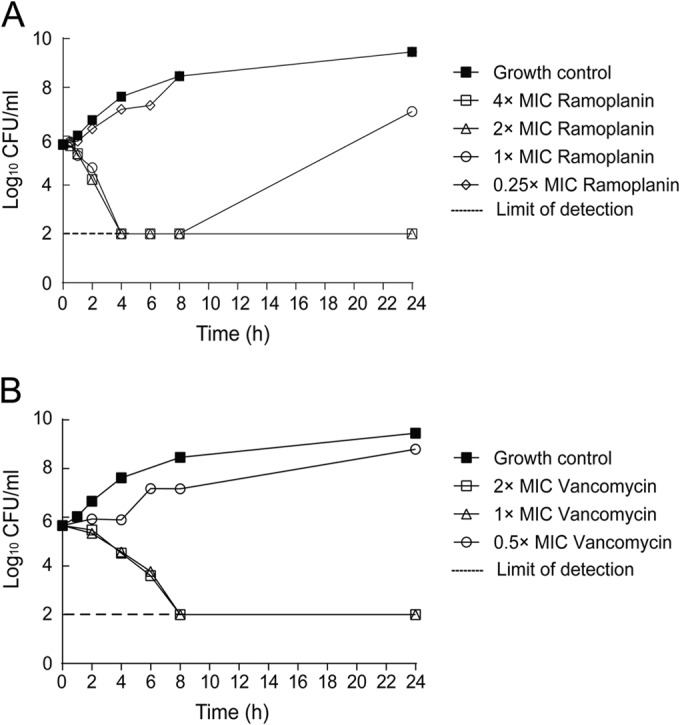
Time-kill curves of MSSA ATCC 25923 exposed to ramoplanin (A) and vancomycin (B) at different concentrations in relation to their respective MICs over a period of 24 h. The growth control contained no antibiotics. The limit of detection was 100 CFU/ml. Mean values of duplicate CFU/ml measurements are plotted.

The MBC of ramoplanin was 2-fold higher than its MIC, which was in agreement with a previous definition of bactericidal agents (45). Time-kill curve analysis of MSSA ATCC 25923 showed rapid bactericidal killing occurred when ramoplanin was present at its MBC and above, as the viable cell counts decreased by approximately 5-fold within 1 h and by at least 1,000-fold (3 log_10_ CFU/ml) in less than 4 h when tested at the MBC and above (Fig. 2A). This was consistent with its reported bactericidal activity against antibiotic-resistant enterococci (12) and MRSA (46). There was a reduction of >3 log_10_ CFU/ml observed when MSSA ATCC 25923 was exposed to vancomycin at the MBC and above for 8 h (Fig. 2B), which was in agreement with results in previous studies (47, 48).

### Ramoplanin binds to membranes in a dose-dependent manner with higher affinity for anionic over zwitterionic membranes.

SPR studies using a Biacore L1 biosensor chip (36, 37, 49) were used to gain insight into interactions of ramoplanin with phospholipid bilayers representing mammalian and bacterial membranes. In this study, the zwitterionic phospholipids POPC and POPE were used to mimic more neutral mammalian cell membranes, while the anionic phospholipid POPG was used in combination with POPC to mimic more anionic bacterial cell membranes (37, 50). The immobilization of phospholipid bilayer on the chip was measured as the changes in resonance units (RU) against time, and the amount of lipid loaded (RU_lipid_) varied depended upon the lipid types: 3,000 RU for POPC:POPG (8:2), 7,000 RU for POPC and 8,000 RU for POPC:POPE (8:2) (see Fig. S1 in the supplemental material). Using the established correlation between RU and absorbed mass (1 RU = 1 pg/mm^2^) (51, 52), the POPC:POPG, POPC, and POPC:POPE membranes had surface densities equivalent to approximate 3, 7, and 8 ng/mm^2^, respectively. These findings lead to the theoretical surface density for a perfect, unilamellar planar phospholipid bilayer of 4.4 ng/mm^2^ (52).

**FIG 3:**
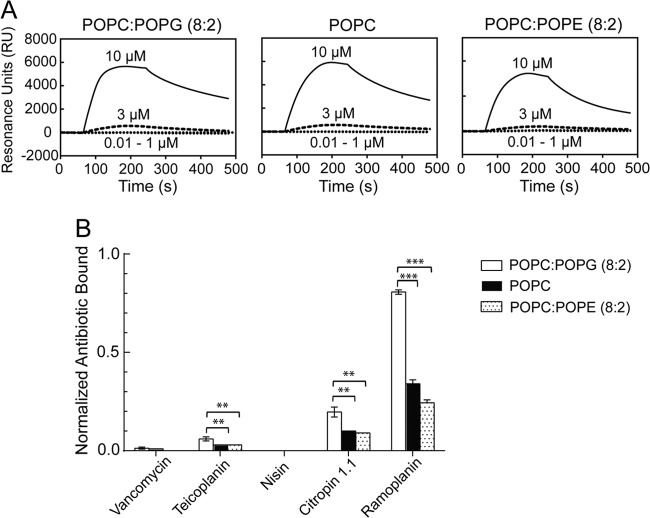
(A) Typical SPR sensorgrams, showing the changes in resonance units (RU) against time upon binding of ramoplanin (0.01 to 10 μM) to lipid bilayers comprised of different lipid mixtures reconstituted on an L1 lipid capture sensor chip. Ramoplanin was injected over the lipid surface for 180 s, and the ramoplanin-lipid complex was then allowed to dissociate for 300 s. The baseline was set to zero for ease of visualization and represents the value RU_Lipid_. (B) Comparison of the binding affinities of antibiotics toward three different lipid bilayers at 10 μM, after normalization of the amount of antibiotic bound (RU_Bound_) in SPR sensorgrams against the corresponding antibiotic molecular weight and the amount of lipid loaded on each channel of the sensor chip (RU_Lipid_), as described in Materials and Methods. Statistical comparisons of normalized antibiotic bound to the anionic (POPC:POPG, 8:2) and zwitterionic (POPC and POPC:POPE, 8:2) membranes was performed by using the two-tailed Student *t* test. **, *P* < 0.01; ***, *P* < 0.001. Data are means ± standard deviations (*n* = 3).

Ramoplanin was then injected across the stable phospholipid bilayers in a series of concentrations ranging from 0.01 to 10 μM, giving rise to the resulting sensorgrams that showed the changes in RU as a function of time upon binding of ramoplanin to the mimetic membranes (RU_Bound_) (Fig. 3A). Low concentrations (0.01 to 1 μM) of ramoplanin showed a negligible association, while high concentrations (3 and 10 μM) showed a significant association with binding responses, up to approximate 6,000 RU for both anionic and zwitterionic membranes. Ramoplanin at concentrations ranging from 0.01 to 1 μM showed complete dissociation after completion of the injection, whereas ramoplanin at concentrations of 3 and 10 μM had a gradual dissociation, with approximately 40% to 60% of material remaining on membranes at 480 s. These data showed that ramoplanin bound to both anionic and zwitterionic membranes in a dose-dependent manner.

Comparison of the binding affinities of ramoplanin toward each of the three different lipid bilayers required normalization of the RU_Bound_ value against the molecular weight and the value RU_Lipid_ for the respective phospholipid bilayer (37), and the values were ranked as follows: POPC:POPG (8:2) > POPC > POPC:POPE (8:2) (Fig. 3B). There was a significant preference for POPC:POPG (8:2) anionic membrane over either zwitterionic membranes (*P* < 0.001). The difference between the two zwitterionic membranes may have resulted from different membrane packing, as the smaller headgroup of POPE compared to POPC can lead to tighter membrane packing (53).

Citropin 1.1, which has been reported to selectively bind to anionic membranes (37), displayed similar membrane specificity to ramoplanin but had significantly reduced binding affinity (Fig. 3B). Nisin showed no binding to either anionic or zwitterionic phospholipid bilayers, which was in agreement with findings of a previous model membrane study (54). Vancomycin displayed negligible binding to each of the three different membranes, consistent with previous SPR study results (36, 37). Teicoplanin bound preferentially to the anionic membrane over zwitterionic membranes, which may potentially be attributed to its fatty acyl chain and amine moieties.

### Ramoplanin causes dose-dependent bacterial membrane depolarization.

**FIG 4:**
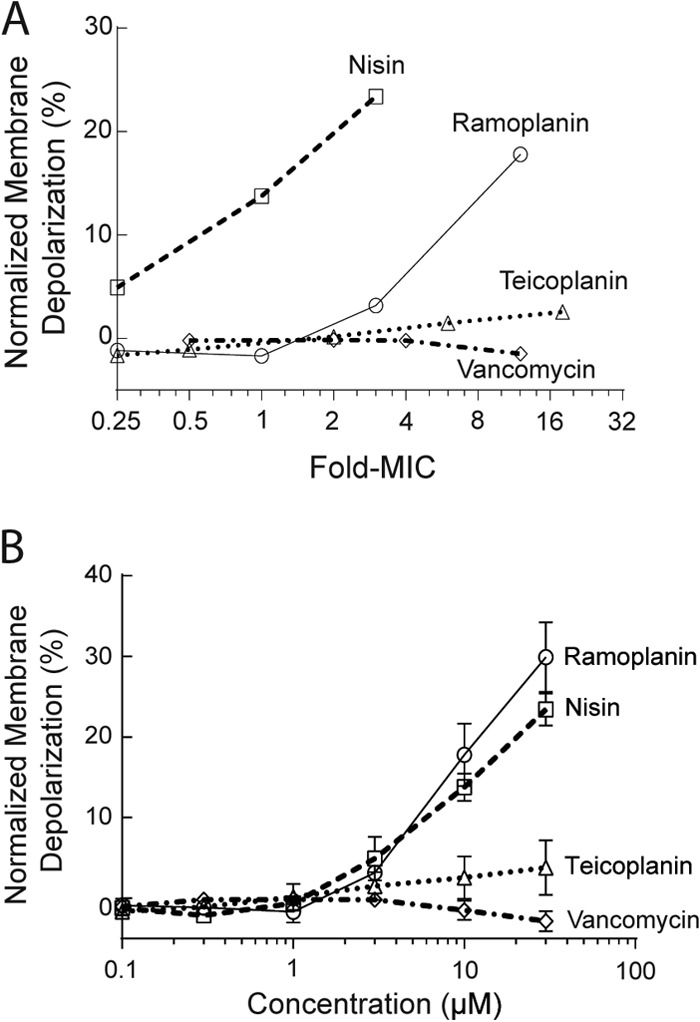
Antibiotic-induced membrane depolarization, demonstrated by the fluorescence leakage from MSSA ATCC 25923 following 30 min of antibiotic treatment, as a function of the fold change for the respective MIC (A) and as a function of the concentration (in μM) (B) against MSSA ATCC 25923, relative to the complete leakage observed for the known pore-forming antibacterial peptide citropin 1.1 at 0.5× its MIC (∼10 μM). Means ± standard deviations are shown (*n* = 3).

Ramoplanin-induced cytoplasmic membrane potential change was determined by measuring the effect of ramoplanin on MSSA ATCC 25923 membrane potential gradient depolarization, using the membrane potential-sensitive dye DiSC_3_(5) (37, 38). In this assay, DiSC_3_(5) localizes in the bacterial membrane according to the intact membrane potential gradient, where the fluorescence is self-quenched (55, 56). Compounds that depolarize the membrane potential gradient release the membrane-bound DiSC_3_(5) into the assay medium, where fluorescence can be measured (56). Citropin 1.1, which rapidly depolarizes cytoplasmic membrane due to pore formation (37), was used as a positive control, whereas vancomycin was used as a negative control, as it does not directly compromise membrane integrity (57). Citropin 1.1 triggered a rapid and complete fluorescence leakage at 0.5× MIC (∼10 μM) and higher concentrations within 30 min (see Fig. S2 in the supplemental material), while no leakage was observed after the addition of vancomycin at 12× MIC for a period of 30 min (Fig. 4), which was consistent with a previous study (37). Nisin was shown to depolarize bacterial membranes at a concentration as low as 0.25× MIC and caused dramatic membrane depolarization at the MIC and above (Fig. 4), which was in agreement with previous study (58, 59). Ramoplanin was shown to cause dose-dependent membrane depolarization but less efficiently than nisin: for ramoplanin, 3× MIC was required to depolarize the cells to a similar extent as 0.25× MIC nisin (Fig. 4). Depolarization induced by ramoplanin was significantly stronger than that of teicoplanin, which triggered measurable but minor fluorescence leakage at concentrations significantly higher than the MIC (Fig. 4).

### Ramoplanin causes morphological changes to the bacterial cell wall and cell membranes.

**FIG 5:**
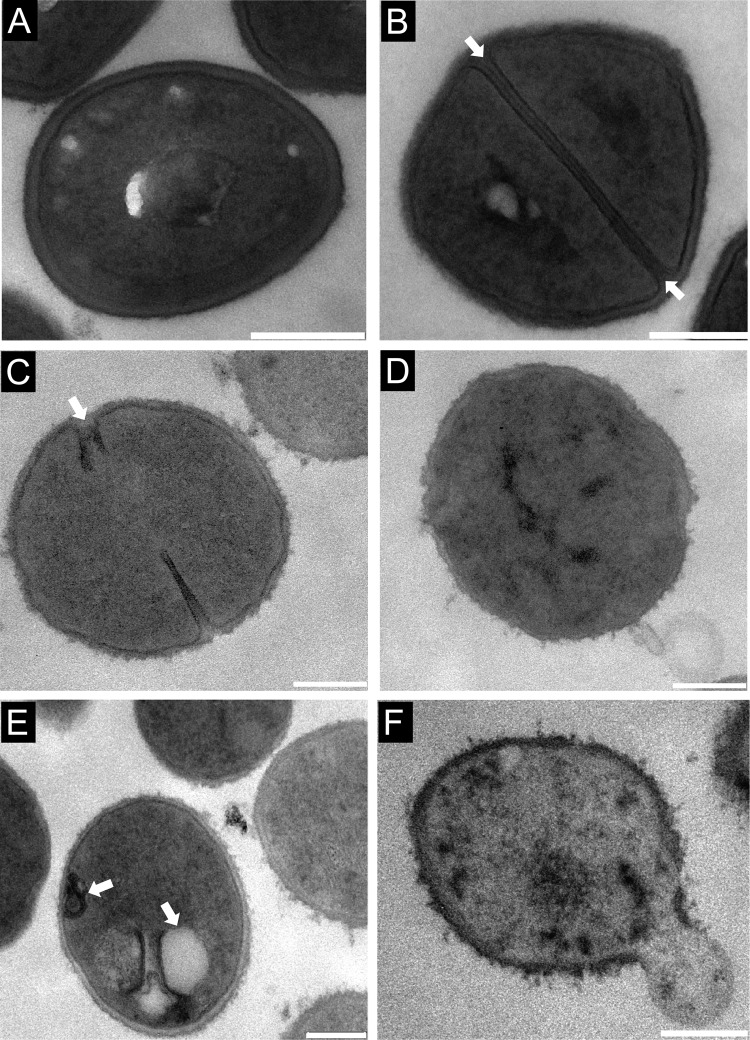
TEM images of MSSA ATCC 25923 at mid-log phase (A and B). Untreated bacteria are spheroidal with a distinct septal midline (arrows in panel B). After incubation with ramoplanin at a concentration of 1× MIC for 3 h, bacteria presented with deformed septa (arrow in panel C), irregular thickening cell wall (D), mesosomes (arrows in panel E), and lysis of the membrane (F), resulting in leakage of cytosolic contents. Bars, 200 nm.

Untreated MSSA ATCC 25923 cells exhibited a normal coccoid shape (circular and smooth), surrounded by a clear and intact cell membrane and cell wall with a uniform thickness of 25 to 30 nm (Fig. 5A) and with a prominent septal midline within the nascent septum (Fig. 5B), consistent with previous microscopy studies (40, 60, 61). The vast majority of MSSA cells exposed to ramoplanin displayed deformed septa that were slightly thickened, misshapen, and lacked distinct septal midlines (Fig. 5C), while exposure to vancomycin did not cause septal deformation or loss of midline (see Fig. S3A in the supplemental material). Ramoplanin-treated MSSA cells also showed irregular thickening and an increase in the occurrence of “fuzzy” cell walls (Fig. 5D), as was the case for vancomycin (see Fig. S3B). In addition to morphological changes to septa and cell walls, ramoplanin elicited cell membrane alterations, demonstrated by the appearance of mesosome structures in cell membranes (Fig. 5E), which were absent in vancomycin-treated MSSA cell membranes. Cytoplasmic contents were further released from the disrupted cells to form ghost cells (Fig. 5F).

## DISCUSSION

Gram-positive bacterial membranes are negatively charged due to the presence of PG and to a lesser extent cardiolipins (28, 29), while mammalian membranes contain a high proportion of zwitterionic phospholipids PE and PC (62). As the PE content of some mammalian cells is around 20%, a POPC:POPE (8:2, wt/wt) mixture was used as a mammalian membrane mimic for SPR studies. Similarly, a POPC:POPG (8:2, wt/wt) mixture was used to mimic a simplified bacterial membrane (29, 63).

Ramoplanin has been proposed to form an amphipathic C_2_ symmetrical dimer that has a membrane binding hydrophobic surface and a hydrophilic surface, in which Lipid II is captured between the two hydrophobic surfaces of the dimer (15). McCafferty and coworkers have reported without experimental details that ramoplanin binds to PE and PG unilamellar vesicles in the absence of Lipid II (26, 27) In this study, SPR was used to show that ramoplanin binds to both anionic and zwitterionic membranes at concentrations of 3 μM and above in a dose-dependent manner, with enhanced selectively for anionic over zwitterionic membranes. Nisin did not bind to any of the membranes in these SPR studies, which was consistent with previous studies that showed nisin specifically bound to the Lipid II pyrophosphate moiety (*K_d_* of 10^−8^ M) rather than phospholipid bilayers (*K_d_* of 10^−4^ M) (54, 64, 65).

The increased binding affinity of ramoplanin for anionic membranes over zwitterionic membranes was similar to that reported for citropin 1.1 (37). The selectivity of citropin 1.1 for anionic membranes has been suggested to be due to the electrostatic interaction of the positively charged (+2) citropin 1.1 with the negatively charged PG headgroups (37, 66). Previous studies have shown that the Orn-10 residue occupies the hydrophobic/hydrophilic interface of the ramoplanin dimer, while the Orn-4 residue lies on the hydrophilic interface (6, 26). Therefore, Orn-10 is in a suitable position to make long-range electrostatic and ion-dipole contacts with the PG headgroups that may be responsible for the membrane selectivity of ramoplanin. The importance of the Orn residues in ramoplanin was demonstrated during an alanine scan study in which the replacement of the Orn-10 residue resulted in a 540-fold loss of activity, while replacement of Orn-4 led to a 44-fold loss in activity (67). Hence, Orn-10 may play a dual role in the mode of action by binding to the Lipid II pyrophosphate moiety (26) and binding preferentially to bacterial membranes.

The hydrophobic acyl chain allows ramoplanin to anchor and insert into the phospholipid bilayer in a transmembrane manner, as is the case for teicoplanin (68, 69), while nisin requires Lipid II to be incorporated into the membrane to trigger membrane insertion. Once bound to the membrane, nisin undergoes Lipid II-induced aggregation that causes membrane disruption and leakage (54). Ramoplanin has been shown to undergo Lipid II-induced aggregation *in vitro* (5, 27, 70), which could also be occurring on the bacterial membrane surface. Both ramoplanin and teicoplanin caused dose-dependent membrane depolarization in a weak detergent-like manner without immediate loss of the entire membrane integrity (Fig. 4), whereas citropin 1.1 triggered a sudden and detergent-like membrane disruption regardless of concentration (see Fig. S2 in the supplemental material), which appeared to adopt the membrane-disruptive (“carpet-like”) mechanism (71).

TEM images of untreated and vancomycin-treated MSSA showed the presence of dividing cells with highly contrasted septal midlines, which result from autolysins that hydrolyze polymers of the nascent cross walls, exposing an electron-dense staining area within the septum (40). Exposure to ramoplanin caused septal deformations, and loss of the septal midline could be due to ramoplanin-induced cell wall biosynthesis inhibition (5, 6) or/and membrane depolarization that could affect autolysin activity, which is important in the regulation of autolysis (40, 72). Additionally, ramoplanin elicited obvious cell membrane changes as indicated by the formation of mesosome structures, which are intracytoplasmic membrane inclusions and have been regarded as the indication of cytoplasmic membrane alteration (38). Since the cytoplasmic membrane is essential for cell wall synthesis and turnover, the cytoplasmic membrane alteration may also affect cell wall integrity (73), as indicated by the irregular thickening and more “fuzzy” cell walls of ramoplanin-treated MSSA.

**FIG 6:**
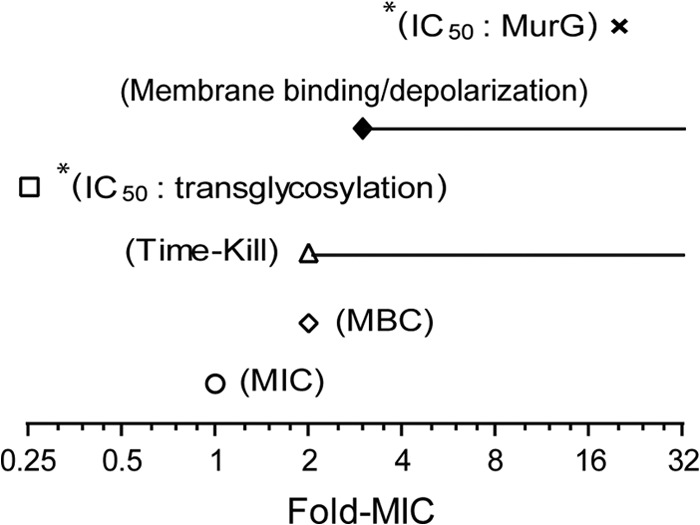
Comparison of ramoplanin antibacterial activity against MSSA ATCC 25923 with mode-of-action parameters. *, data are from the published literature (other data are from this study). Inhibition of the MurG-catalyzed reaction of peptidoglycan biosynthesis has been reported to occur at a concentration significantly higher than the MIC (IC_50_, 20× MIC against MSSA ATCC 25923), while ramoplanin inhibits transglycosylation at the concentration correlated with the MIC (IC_5_, 0.25× MIC against MSSA ATCC 25923) (22). Ramoplanin binds to cell membranes and induces membrane depolarization at concentrations close to or above the MBC, which corresponded to its bactericidal concentrations against MSSA observed in time-kill studies.

Ramoplanin inhibits the transglycosylation step of peptidoglycan biosynthesis, with a 50% inhibitory concentration (IC_50_) of 0.25× MIC (Fig. 6) (22), suggesting that transglycosylation inhibition is the primary mode of action of ramoplanin. In this study, ramoplanin was shown to trigger rapid dose-dependent membrane depolarization at concentrations of 3× MIC and above, which was close to its MBC of 2× MIC against MSSA ATCC 25923 (Fig. 6). Time-kill studies indicated that ramoplanin exhibited rapid bactericidal activity at concentrations equal to or above the MBC, producing a 5-fold decrease in viability within 1 h and bactericidal activity (≥9%) in less than 4 h.

The importance of the membrane depolarization and bactericidal killing *in vivo* is difficult to quantify due to the limited published pharmacokinetic (PK) studies (18, 19), as well as its nonsystemic oral administration in human clinical trials. In addition, MIC determinations can be highly dependent on plate type, serum, and BSA (18, 41). The first reported PK properties of ramoplanin were published in 1986, when a daily 10-mg/kg of body weight intravenous (i.v.) dosing in rabbits of ramoplanin alone and ramoplanin with penicillin resulted in maximal ramoplanin plasma levels (*C*_max_) of 28 μg/ml (11 μM; 14× MIC) and a 12-h concentration (C_12_) of 6.4 μg/ml (2.5 μM; 3× MIC) (19) when using the agar dilution method. The ramoplanin MIC in this study against two VRE strains was 0.5 μg/ml and the addition of 10% serum increased the MIC to 2 μg/ml (19). In a recent publication a 20-mg/kg i.v. dosing of ramoplanin in rats gave a *C*_max_ of 79 μg/ml (31 μM; 39× MIC) and a C_12_ of 2 μg/ml (0.8 μM; 1× MIC) using the agar dilution method (18). This study also reported that the MIC of ramoplanin was not affected by the addition 50% human serum for MSSA and MRSA strains (18). In our study, the ramoplanin MIC against MSSA ATCC 25923 in the presence of 50% human serum lowered the MIC from 2 to 0.5 μg/ml (see Table S1 in the supplemental material). The initial concentrations of ramoplanin in *in vivo* studies (18, 19) were above levels in which the *in vitro* ramoplanin-induced membrane depolarization and bactericidal killing can occur, while the presence of serum did not adversely the MICs for staphylococci. These data suggest that there is potential clinical relevance for ramoplanin-induced membrane depolarization and that this depolarization could contribute to the characteristic rapid bactericidal activity of ramoplanin.

## Supplementary Material

Supplemental material
