# Supplementary material for: Ramoplanin at Bactericidal Concentrations Induces Bacterial Membrane Depolarization in Staphylococcus aureus
Source: Antimicrob Agents Chemother. 2014 Nov;58(11):6819–27. doi: 10.1128/AAC.00061-14 (PMC4249368; doi:10.1128/AAC.00061-14)
Supplement: Supplemental material [file supp_58_11_6819__index.html]

Ramoplanin at Bactericidal Concentrations Induces Bacterial Membrane Depolarization in Staphylococcus aureus — Supplemental material 

# Ramoplanin at Bactericidal Concentrations Induces Bacterial Membrane Depolarization in Staphylococcus aureus

## Supplemental material

**Files in this Data Supplement:**

- Supplemental file 1 -

  Supplemental Table S1 and Figures S1 to S3.

  PDF, 256K
