# Supplementary material for: Ramoplanin at Bactericidal Concentrations Induces Bacterial Membrane Depolarization in Staphylococcus aureus
Source: Antimicrob Agents Chemother. 2014 Nov;58(11):6819–27. doi: 10.1128/AAC.00061-14 (PMC4249368; doi:10.1128/AAC.00061-14)
Supplement: Supplemental material [file AAC.00061-14_zac011143428so1.pdf]

Ramoplanin induces bacterial membrane depolarization in *Staphylococcus aureus* at bactericidal concentrations

Mu Cheng, Johnny X. Huang, Soumya Ramu, Mark S. Butler, Matthew. A Cooper*

Institute for Molecular Bioscience, The University of Queensland, Brisbane, Queensland, 4072, Australia

Supplemental material

Table S1 Vancomycin, ramoplanin and teicoplanin MIC and MBC[a] against *S. aureus* ATCC 25923 in MHB media in the presence and absence of Human serum (10% and 50%) in Corning NonTC microtitre plates (CLS3370)

Compound	MHB (μg/ml)		10% Human serum (μg/ml)		50% Human serum (μg/ml)	
	MIC	MBC	MIC	MBC	MIC	MBC
Vancomycin	2	2	1/2	2	2	2
Ramoplanin	2	8/2	0.5/0.25	1/0.5	0.5/0.25	0.5
Teicoplanin	2	4	0.25/0.125	0.25	0.5/0.25	0.5

[a] The MBCs were determined by plating out the clear wells onto an agar plate and were incubated at 37°C overnight. Each colony represents one viable bacterium.

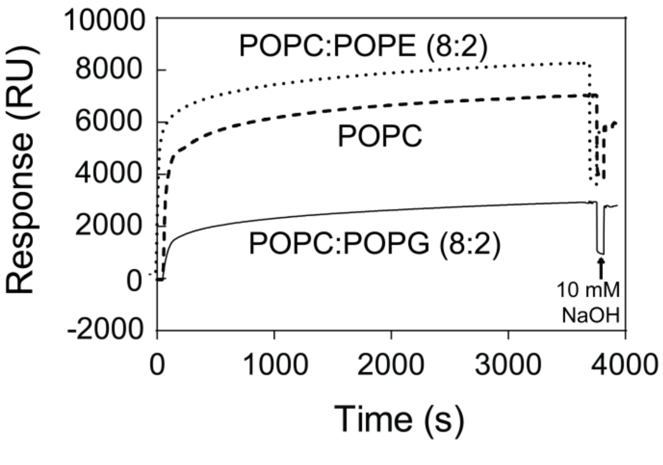

FIG S1 Loading of the Biacore L1 sensor chip with small, unilamellar vesicles comprised of 20 wt% POPG or POPE mixed in POPC, or pure POPC after exposure 3600 s, followed by sequential injection of 10 mM NaOH for 60 s. PBS was used as running buffer.

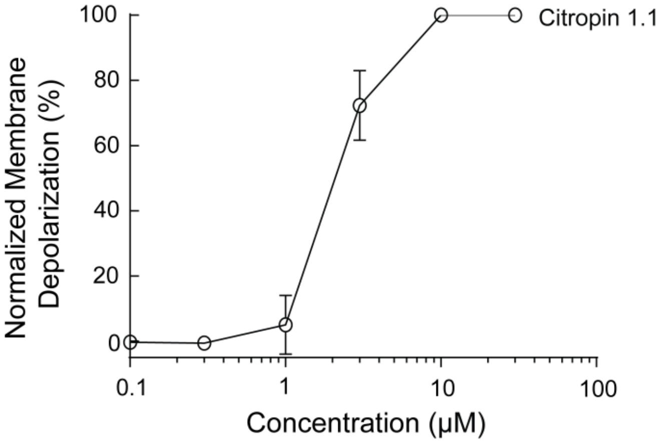

FIG S2 Exposure to citropin 1.1 at concentrations of 10 and 30 μM (~ 0.5× and 1.5× MIC against MSSA ATCC 25923) triggers complete and stable membrane permeability against MSSA ATCC 25923 within 30 min. Mean ± SD for n=3.

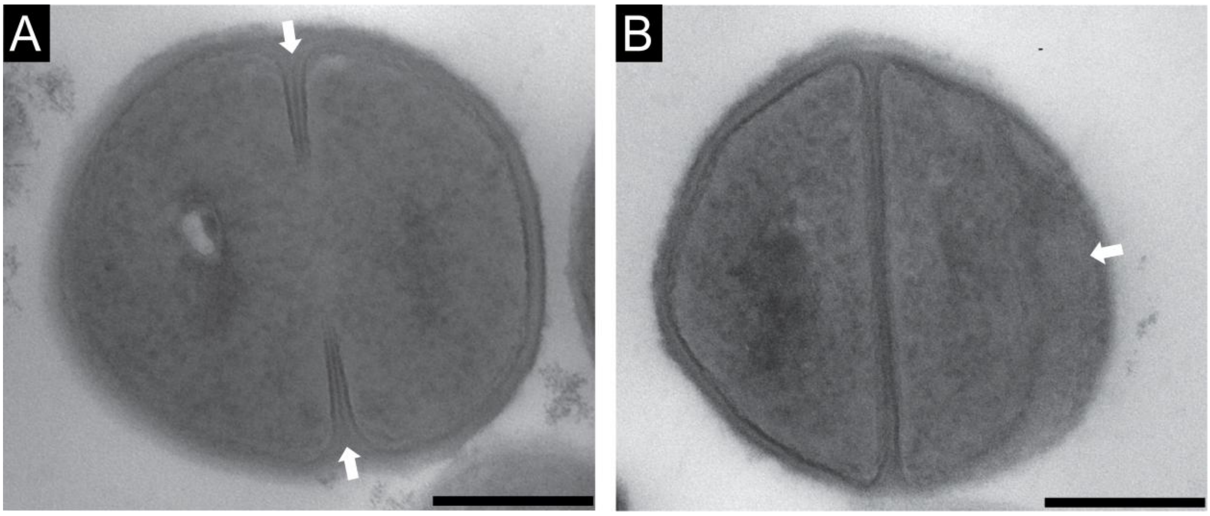

FIG S3 TEM images of MSSA ATCC 25923 at mid-log phase treated with vancomycin at concentration of 16× the MIC for 3 h show normal septa (arrows in A) and a thickened cell wall (arrow in B).
